# Supplementary material for: Adverse events following COVID-19 vaccination in Kwara State, North-central Nigeria
Source: PLOS Glob Public Health. 2022 Aug 15;2(8):e0000835. doi: 10.1371/journal.pgph.0000835 (PMC10021916; doi:10.1371/journal.pgph.0000835)
Supplement: Attachment — Submitted filename: RESPONSE TO REVIEWERS.docx [file pgph.0000835.s002.docx]

Abram Luther Wagner, PhD, MPH
Academic Editor
PLOS Global Public Health

June 4, 2022

Dear Dr. Wagner,

Thank you for inviting us to submit a revised draft of our manuscript entitled, “Adverse Events Following COVID-19 Vaccination in Kwara state, North Central Nigeria” to PLOS Global Public Health. We also appreciate the time and effort you and each of the reviewers have dedicated to providing insightful feedback on ways to strengthen our paper. Thus, it is with great pleasure that we resubmit our article for further consideration. We have incorporated changes that reflect the detailed suggestions you have graciously provided. We also hope that our edits and the responses we provide below satisfactorily address all the issues and concerns you and the reviewers have noted.

To facilitate your review of our revisions, the following is a point-by-point response to the questions and comments delivered in your letter dated May 4, 2022.

**EDITOR SUGGESTIONS:**

1. We have amended your Competing Interest statement to comply with journal style. We kindly ask that you double-check the statement and let us know if anything is incorrect.
   - **RESPONSE:** The Competing Interest statement has been checked but no amendment was noticed. The statement is however correct in its present form.
2. We ask that a manuscript source file is provided at Revision. Please upload your manuscript file as a .doc, .docx, .rtf

- **RESPONSE:** The manuscript file is uploaded as a .doc

3. We have noticed that you have uploaded Supporting Information files, but you have not included a list of legends. Please add a full list of legends for your Supporting Information files after the references list.
   - **RESPONSE:** The list of legends for the Supporting Information files is provided after the references list. It is named S1 Dataset.
4. Please provide an Author Summary. This should appear in your manuscript between the Abstract (if applicable) and the Introduction and should be 150–200 words long.

- **RESPONSE**: The author summary has been provided between the abstract and the introduction

5. Please review your reference list to ensure that it is complete and correct. Any changes to the reference list should be mentioned in the rebuttal letter that accompanies your revised manuscript.

- RESPONSE: The reference list has been updated to accommodate new additions.

**REVIEWER 1 COMMENTS:**

6. This is a well-written paper, and the work will contribute to the important area of vaccine safety reporting and the role it plays in mitigating vaccine hesitancy.
   - **RESPONSE:** Thank you so much for your time and energy committed to this review. Your comments are valuable and have improved this work.
7. In the methods, there is no mention of which vaccine(s) is/are being monitored but paragraph 2 of the discussion talks about AstraZeneca and Morderna. Authors need to clarify on which vaccine to which the AEFIs are attributed.
   - **RESPONSE:** Thank you for this suggestion. The name of the covid vaccine received (AstraZeneca) has been mentioned in the method section of both the abstract and the main manuscript. Also the Moderna vaccine mentioned in paragraph 2 of the discussion has been clarified as another type of vaccine investigated by another study.
8. Was this a document review of records at the health facilities? Or the vaccine recipients were interviewed using a dedicated tool by health worker or research assistant?

- **RESPONSE:** This research was a study of the reported adverse events to AstraZeneca COVID vaccination at the mentioned facilities. At the point of vaccination, recipients were informed to report any adverse events following the vaccination to the vaccine centers or emergency room depending on the severity of the adverse event. Those who reported adverse events were monitored by twice-daily phone calls and home visits and were followed up for 3months after vaccination. Kindly see the last few statements of the study design section of the method.

9. Table 4 and 5: We need to be careful when interpreting the chi-square and p-values because there are many cells with zero or n<5. Did authors use Fisher’s exact test?

- **RESPONSE:** Thank you for this suggestion. The tables were reviewed as follows: The Pearson Chi square test and Fisher’s exact test were used to determine the association when none and greater than 20% of the expected counts respectively were less than 5 for 2x2 tables. While Pearson Chi-square test and likelihood ratio test were used to determine the association when none and greater than 20% of the expected counts respectively were less than 5 for larger than 2x2 tables.

10. Need to be consistent with the use of ‘adverse events’, ‘adverse reactions’, ‘negative reactions’ and ‘side effects.’ They are used interchangeably, and authors need to harmonise that.

- **RESPONSE:** Thank you for this suggestion. The write-up has been reviewed with consistent use of the term “adverse events” throughout the write-up.

11. Paragraph 3 of discussion is confusing. The reasons given for fewer numbers are .......general population and being community-based health care facilities..... These reasons may not be convincing to the general audience. I think the AE reporting culture may be one of the reasons …. Please explore further to make your argument stronger.

- **RESPONSE**: This part has been modified with the addition of the following “Poor reporting culture of adverse events may be another reason for the disparity. Although low reporting of adverse events is a global phenomenon, the Nigerian situation is worse. Despite the crucial role of patients in spontaneous reporting of adverse events, there exists a general belief in Nigeria’s traditional communities that adverse events are proof of the effectiveness of medicinal agents.”

12. Discussion Para 4: what do you mean by this statement ‘……If age is a factor of AE, there is no clear consensus.’ A lot of contradicting information from sentence number 4 onwards.

- **RESPONSE:** This section has been modified and the contradicting information has been sorted out.

13. Discussion Para 6: the last sentence seems to be incomplete.

- **RESPONSE:** The last sentence has been completed with modification.

14. Discussion Para 7: the last 2 sentences may be seen as subjective because the follow-up time is not mentioned anywhere yet….
    - **RESPONSE:** The sentences were modified as follows: “There was no evidence of blood clots, pulmonary embolism, deep vein thrombosis, focal neurological deficit, or cardiac event in this study's participants within 3 months of follow-up. However, the duration of follow-up may not permit definitive comment on the long-term adverse event of the vaccine”. The follow-up period of 3 months has also been mentioned in the method section of this write-up.

**REVIEWER 2 COMMENTS:**

15. Reviewer 2 general comment: The author described the fear of adverse effects of vaccination in general public resulting hesitancy and resistance of vaccination.
    - **RESPONSE:** Thank you so much for committing your time and energy to review our work and also for your valuable comments.
16. Moreover the lower number are been vaccinated in the particular state.
    - **RESPONSE:** The number of the vaccinated individuals quoted was not the total number vaccinated in the whole state but the total number of vaccine recipients in the 5 centers where the study was carried out as stated in the method section.
17. The evaluation was done by taking only one vaccine i.e. AstraZeneca, what about other vaccines side effects/AE?.

- **RESPONSE:** Throughout the study period, AstraZeneca COVID-19 vaccine was the only available vaccine in the state, hence, it was the only one administered. Advert events of other vaccines could not be studied or compared to that of AstraZeneca because they were not available for administration. Kindly see the method section.

18. The interventions made in case of fatal AE that involved hospitalization were not clearly described the outcomes. What kind of observations made in interventions?
    - **RESPONSE:** The 30-year old woman who had anaphylactic shock was managed as such. We felt a detailed description of the management of the case is beyond the scope of this article
19. The article is well written, describing some points that should be helpful for considerations in future studies. However, it is important to compare the data on severity of adverse effects of one or more vaccines that have been used in the state.

- **RESPONSE:** Thank you much for this comment. As noted in response to comment number 17 above, the AstraZeneca COVID-19 vaccine was the only administered vaccine in the state during the study period. However, this is a very good recommendation for subsequent research.

**CONCLUDING REMARKS**: Again, thank you for giving us the opportunity to strengthen our manuscript with your valuable comments and queries. We have worked hard to incorporate your feedback and hope that these revisions persuade you to accept our submission.

Sincerely,

Dr Yahkub Babatunde Mutalub

(Corresponding Author)

Department of Clinical Pharmacology

College of Medical Sciences

Abubakar Tafawa Balewa University

PMB 0284 Bauchi Nigeria

Tel +2348032073874

Email bymutalub@atbu.edu.ng
