# Supplementary material for: Adverse events following COVID-19 vaccination in Kwara State, North-central Nigeria
Source: PLOS Glob Public Health. 2022 Aug 15;2(8):e0000835. doi: 10.1371/journal.pgph.0000835 (PMC10021916; doi:10.1371/journal.pgph.0000835)
Supplement: Attachment — Submitted filename: RESPONSE TO REVIEWERS.docx [file pgph.0000835.s003.docx]

Abram Luther Wagner, PhD, MPH
Academic Editor
PLOS Global Public Health

June 30, 2022

Dear Dr. Wagner,

Thank you for inviting us to submit a revised draft of our manuscript entitled, “Adverse Events Following COVID-19 Vaccination in Kwara state, North Central Nigeria” to PLOS Global Public Health. We also appreciate the time and effort you and each of the reviewers have dedicated to providing insightful feedback on ways to strengthen our paper. Thus, it is with great pleasure that we resubmit our article for further consideration. We have incorporated changes that reflect the detailed suggestions you have graciously provided. We also hope that our edits and the responses we provide below satisfactorily address all the issues and concerns you have noted.

To facilitate your review of our revisions, the following is a point-by-point response to the questions and comments delivered in your letter dated June 23, 2022.

**EDITOR SUGGESTIONS:**

1. Could you separate the first sentence of the abstract into two sentences?
   - **RESPONSE:** The first sentence of the abstract has been separated into two as follows: ‘Safe and effective vaccination remains the mainstay of control of COVID-19 because there is still no universally recommended treatment. This strategy is however being threatened by vaccine hesitancy and resistance due to fear of adverse events and safety concerns”.
2. For the discussion first two sentences - could you reword? I would hesitate to say that vaccination is the most efficient approach to illness prevention. (you could say "vaccination is one of the most efficient approaches to illness prevention").

- **RESPONSE:** The sentence has been reworded as “ Vaccination is, therefore, one of the most efficient approaches to illness prevention”

3. You focus in the discussion on the difference in AE incidence between males and females. However, I don't think you found a significantly different result. If you really want to discuss this, I would start the paragraph saying something like "We found a non-significantly higher proportion of AEs in woman than men. [other studies...]"
   - **RESPONSE:** The section in the discussion which compares adverse events between males and females has been expunged.
4. I would also hesitate to make comparisons by demographic group. Your Table 1 is good, but to adequately compare the risk of AE, we would need to know what these numbers are in the total 5816 individuals who were vaccinated. That is to say, I would recommend not focusing much on age or gender - you could keep those paragraphs in discussion or delete them. Your discussion about the types of AEs was more interesting and relevant given your data.

- **RESPONSE**: The section in the discussion which compares adverse events among age groups has been expunged.

5. You note a very low proportion of AEs in your study. Your description seems adequate, but I would move it to the limitations section.

- RESPONSE: The poor adverse event reporting culture among Nigerians which may likely account for the very low proportion of reported adverse events has been added to the limitation section.

6. The reference list has been updated to reflect the expunged information

**CONCLUDING REMARKS**: Again, thank you for giving us the opportunity to strengthen our manuscript with your valuable comments. We have worked hard to incorporate your feedback and hope that these revisions persuade you to accept our submission.

Sincerely,

Dr Yahkub Babatunde Mutalub

(Corresponding Author)

Department of Clinical Pharmacology

College of Medical Sciences

Abubakar Tafawa Balewa University

PMB 0284 Bauchi Nigeria

Tel +2348032073874

Email bymutalub@atbu.edu.ng
